# GOPHER: Generator Of Probes for capture Hi-C Experiments at high Resolution

**DOI:** 10.1186/s12864-018-5376-4

**Published:** 2019-01-14

**Authors:** Peter Hansen, Salaheddine Ali, Hannah Blau, Daniel Danis, Jochen Hecht, Uwe Kornak, Darío G. Lupiáñez, Stefan Mundlos, Robin Steinhaus, Peter N. Robinson

**Affiliations:** 1 Institute of Medical Genetics and Human Genetics, Charité-Universitätsmedizin Berlin, Augustenburger Platz 1, Berlin, 13353 Germany; 2 Max Planck Institute for Molecular Genetics, Ihnestr. 63-73, Berlin, 14195 Germany; 3 The Jackson Laboratory for Genomic Medicine, 10 Discovery Drive, Farmington, 06032 CT United States; 4 Genomics Unit, Centre for Genomic Regulation, Carrer del Dr. Aiguader 88, Barcelona, 08003 Spain; 5 Berlin Brandenburg Center for Regenerative Therapies (BCRT), Charité-Universitätsmedizin Berlin, Augustenburger Platz 1, Berlin, 13353 Germany; 6 Epigenetics and Sex Development Group, Berlin Institute for Medical Systems Biology, Max-Delbrück Center for Molecular Medicine, Berlin-Buch, 13125 Germany; 7 Institute for Systems Genomics, University of Connecticut, Farmington, 06032 CT United States

**Keywords:** Gene regulation, Nuclear organization, Promoter-enhancer interactions, Capture Hi-C, Java

## Abstract

**Background:**

Target enrichment combined with chromosome conformation capturing methodologies such as capture Hi-C (CHC) can be used to investigate spatial layouts of genomic regions with high resolution and at scalable costs. A common application of CHC is the investigation of regulatory elements that are in contact with promoters, but CHC can be used for a range of other applications. Therefore, probe design for CHC needs to be adapted to experimental needs, but no flexible tool is currently available for this purpose.

**Results:**

We present a Java desktop application called GOPHER (Generator Of Probes for capture Hi-C Experiments at high Resolution) that implements three strategies for CHC probe design. GOPHER’s simple approach is similar to the probe design of previous approaches that employ CHC to investigate all promoters, with one probe being placed at each margin of a single digest that overlaps the transcription start site (TSS) of each promoter. GOPHER’s simple-patched approach extends this methodology with a heuristic that improves coverage of viewpoints in which the TSS is located near to one of the boundaries of the digest. GOPHER’s extended approach is intended mainly for focused investigations of smaller gene sets. GOPHER can also be used to design probes for regions other than TSS such as GWAS hits or large blocks of genomic sequence. GOPHER additionally provides a number of features that allow users to visualize and edit viewpoints, and outputs a range of files useful for documentation, ordering probes, and downstream analysis.

**Conclusion:**

GOPHER is an easy-to-use and robust desktop application for CHC probe design. Source code and a precompiled executable can be downloaded from the GOPHER GitHub page at https://github.com/TheJacksonLaboratory/Gopher.

**Electronic supplementary material:**

The online version of this article (doi:10.1186/s12864-018-5376-4) contains supplementary material, which is available to authorized users.

## Background

Functional elements that are widely separated in the linear sequence of the genome can be brought into contact with one another by the folding of the genome in three-dimensional space. A series of extensions of the original targeted chromosome conformation capture (3C) method that was introduced in 2002 [1] culminated in Hi-C, a global method for interrogating chromatin interactions that combines formaldehyde-mediated cross-linking of chromatin with fragmentation, DNA ligation, and high-throughput sequencing to characterize interacting loci on a genome-wide scale [2]. Hi-C has been used to investigate the large scale organizational architecture of the genome, revealing the existence of megabase-sized local chromatin interaction domains termed topologically associating domains (TADs) [3]. Owing to the complexity of Hi-C libraries, it is not feasible to investigate interactions between specific gene promoters and their distal regulatory elements. For instance, roughly 100 million reads are required to obtain 40kb resolution [4]. Given that a linear increase of resolution requires a quadratic increase in total sequencing depth [5], obtaining the 5kb or better resolution that is desirable for investigating individual promoter-enhancer interactions would be costly. Recently, capture Hi-C (CHC) and capture-C methodologies were developed as alternative approaches to overcome these difficulties. These techniques employ a hybridization technology similar to exome capture that enriches Hi-C libraries for viewpoint sequences representing loci of interest using biotinylated cRNA probes.

CHC has been used in a variety of experimental settings to provide more in-depth data for specific loci than would be feasible with Hi-C. For example, promoter CHC focuses on the enrichment of gene promoters in order to identify functional interactions with distal regulatory elements such as enhancers [6–10]. Other applications include the investigation of the potential regulatory effects of disease-associated single nucleotide polymorphisms (SNPs) identified by genome-wide association studies (GWAS). Of note, the majority of these so-called GWAS hits are located in non-coding and likely regulatory sequences, whose effects are, in the lack of further evidence, commonly assigned to the nearest gene. CHC has suggested the inaccuracy of these assumptions by showing that some distal interactions are associated with stronger effects on expression than interactions with neighboring genes, thereby providing strong evidence that altered regulation of a distal gene underlies the mechanism of certain GWAS hits [11–18]. In particular, one study on 1999 SNPs associated with cardiovascular disease revealed that more than 90% of the SNP-target gene interactions did not involve the nearest neighbor, and 40% of the SNPs displayed interactions with two or more genes [19], demonstrating the value of CHC for understanding disease biology.

CHC has also been used to analyze gene regulation programs in differentiation and disease [20–22] by profiling interactions across large genomic regions and by characterizing the effects of structural variation on chromatin organization. For instance, one study investigated the effects of genomic duplications on the TAD architecture of the genome using CHC and 4C-seq methods, and showed that duplications can result in the formation of new chromatin domains (neo-TADs) with pathologic alterations of gene regulation [23].

CHC employs a set of biotinylated oligonucleotides that are designed to hybridize to and ’capture’ target sequences; such oligonucleotides are usually referred to as baits or probes. Several technologies are commercially available for capture of exonic sequences in exome sequencing [24]. These methods can be adapted for CHC by means of a custom design for probes that hybridize to promoter sequences or other desired CHC target regions. Because of the diversity of CHC applications, users are faced with the challenge of designing probes for specific experimental settings.

To our knowledge, only two tools are available for capture Hi-C probe design. CapSequm [24] is a web application that can be readily used thorough a web browser, but the number of viewpoints is limited to 1000 viewpoints at a time. HiCapTools [25] overcomes this limitation, but is a command-line tool that needs to be compiled from source. Both CapSequm and HiCapTools implement an approach to probe design similar to what we call the ’simple approach’ in this manuscript, and do not implement features that would be required to design probes according to the simple-patched and extended strategies that we introduce in this manuscript.

Here we present GOPHER (*Generator Of Probes for capture Hi-C Experiments at high Resolution*), an easy-to-use Java-based desktop application that provides a suite of methods and visualization tools for the automated design and subsequent manual curation of viewpoints. GOPHER enables all steps required for probe design to be performed in a unified framework that leads users from the download of the genome, alignability, and transcript files, through the choice of parameters such as target genes or regions, restriction enzymes, and desired thresholds for GC content, alignability, and digest length. Users can inspect the genomic context of each of the generated viewpoints, and can add or remove digests (restriction fragments) if desired. GOPHER implements three main approaches to probe design, including two that have not previously been available. GOPHER outputs a series of files including a probe file that can be used to order probes (baits) for the enrichment of the targeted regions in capture Hi-C experiments. Additionally, summary statistics are generated that can be used for documentation of the final design. Users can generate a digest file containing attributes of the selected and unselected digests relevant for downstream analysis.

## Results

We present an easy-to-use software application for the design of CHC probes that uses one of three approaches and allows users to set a wide range of parameters for different experimental situations. GOPHER implements three main strategies for probe design. The simple approach generates probes that are similar to those used for many previously published capture Hi-C studies: One digest is selected for each target region (often including a transcription start site of a gene), and two probes are placed at the outermost ends of the digest. The simple-patched approach “patches” viewpoints that are poorly covered by single digests. GOPHER additionally implements a new approach to probe design that we term extended, which is intended to provide greater resolution than the simple approach by performing restriction digestion with a 4-cutter instead of 6-cutter and selecting sets of multiple fragments per target region. In general, the simple and simple-patched approaches are best suited for investigations of larger numbers of targets such as a promoterome in which all promoters of all coding genes are investigated [7, 8, 10], whereas the extended approach is more suited to investigate smaller numbers of genes (e.g., 500–1000) involved in a biological process of interest [6, 24, 26]. All approaches are also suitable for other categories of target regions such as GWAS hits or larger blocks of genomic sequence.

### Data preparation and parameter settings

**Fig. 1:**
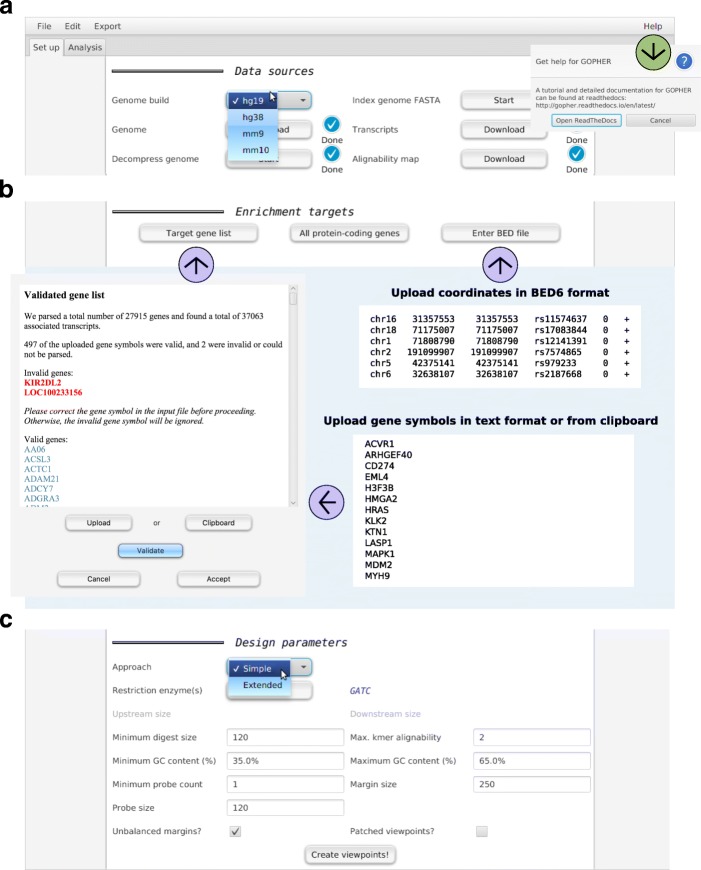
Data preparation and parameter settings. The Setup tab provides an graphical user interface that allows all data and parameters to be collected as required for the creation of viewpoints. (**a**) The upper part of the tab can be used to download and preprocess genome sequence and transcript annotation data for various mouse and human genome builds. (**b**) The middle part can be used to enter the targets for enrichment. Lists of target gene symbols can be uploaded from a text file or from the clipboard. Invalid or outdated gene symbols will be reported so they can be corrected. Alternatively, all protein-coding genes can be selected, or arbitrary genomic positions (such as GWAS hits) can be uploaded in BED6 format. (**c**) The lower part of the Setup tab can be used to specify parameters for probe and digest selection (Table 1 and Fig. 2) using the simple or extended approach

In order to design CHC probes, users need to download and preprocess a substantial amount of sequence and annotation data. GOPHER provides a graphical user interface (GUI) to streamline these tasks (Fig. 1a). Various genome builds for human and mouse can be selected from a drop-down menu, and downloading, unzipping and indexing of genome sequences can be performed with no software requirements other than a Java virtual machine (version 1.8). Furthermore, associated annotation data for transcription start sites and alignability are downloaded and parsed directly from the application. The progress of time-consuming steps such as indexing the genome file is indicated in the GUI. These steps have to be performed only once for a given genome build.

**Table 1:** GOPHER parameters: The users may chose parameter settings that influence the design of probes and digests. In addition, approach-specific parameters can be chosen

Probe parameters	
Probe length	*Explanation:* Length of probes.
	*Default:* 120 bp
Minimum GC content	*Explanation:* The minimum proportion of G and C nucleotides.
	*Default:* 35%
Maximum GC content	*Explanation:* The maximum proportion of G and C nucleotides.
	*Default:* 65%
Alignability	*Explanation:* Maximum mean 50mer alignability.
	*Default:* 2
Digest parameters	
Margin size	*Explanation:* Width of the outermost ends of digests that will be tiled with probes.
	*Default:* 250 bp
Minimum digest size	*Explanation:* Smaller digests cannot be selected.
	*Default:* 120 bp
Minimum number of probes	*Explanation:* At least this number of probes have to be placed in each margin of a balanced digest. The total number of probes in both margins of unbalanced digest must be at least twice this value.
	*Default:* 1
Allow unbalanced margins	*Explanation:* Digest with unequal numbers of probes in each margin are selected during viewpoint creation.
	*Default:* False
Simple parameters	
Allow patching	*Explanation:* Digests that are not well centered at the TSS will be patched during viewpoint creation.
	*Default:* False
Extended parameters	
Maximum distance upstream	*Explanation:* Extension of the viewpoint in upstream direction
	*Default:* 5000 bp
Maximum distance downstream	*Explanation:* Extension of the viewpoint in downstream direction.
	*Default:* 1500 bp

Following this, users specify the desired enrichment targets (Fig. 1b). For promoter CHC, gene symbols can be entered either from a text file or from the clipboard. GOPHER creates one viewpoint for all transcription start sites associated with the entered gene symbols. If gene symbols are used that do not occur in the downloaded annotation data, as can be the case if an invalid or outdated symbol is used (e.g., *P53* instead of the official gene symbol *TP53*), GOPHER will issue a warning and report a list of unmappable symbols that can be used to search for the current correct symbols. An alternative shortcut option allows promoters of all protein coding genes to be selected as targets. GOPHER also accepts a BED file with genomic positions. For instance, the coordinates of GWAS hits can be uploaded in BED6 format.

GOPHER allows the user to set a number of parameters that control the choice of viewpoints, digests, and probes (Table 1) using a graphical user interface (Fig. 1c). In the following sections, we describe how to choose parameters and how to visualize and edit viewpoints.

### Selection of capture Hi-C probes and digests

**Fig. 2:**
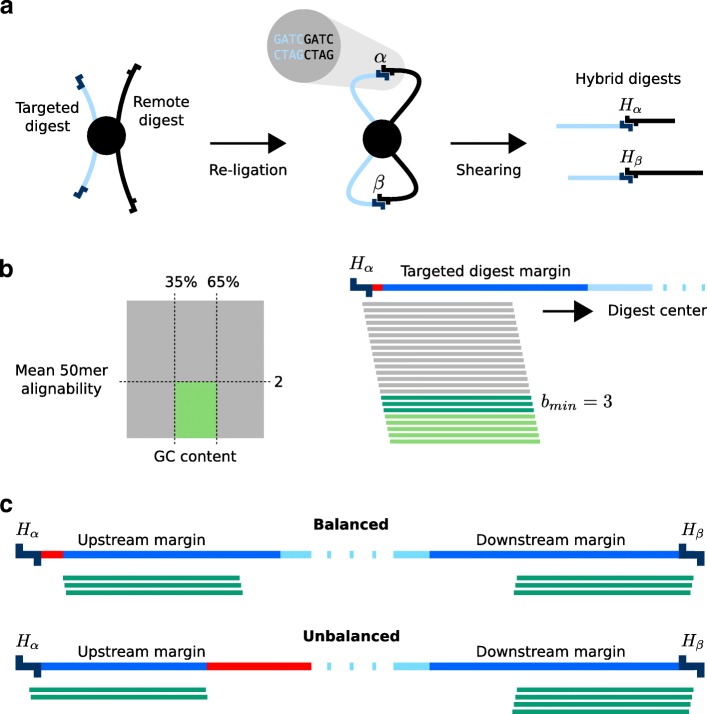
Selection of probes and digests. (**a**) Idealized example of two cross-linked digests from a targeted region (light blue) and a remote interacting region (black). Re-ligation and shearing results in two hybrid digests *H*_*α*_ and *H*_*β*_ consisting of DNA from the targeted and a remote region. (**b**) We assume that the average length of the two parts corresponds to half of the average fragment length of sheared DNA in total. Therefore, only the margins of digests are defined to be target regions (blue). By default, GOPHER uses a margin size of 250 bp. For selection of usable probes only the uniqueness (alignability) of the probe sequence and GC content are taken into account. By default, usable probes are defined as those that have a mean 50mer alignability ≤ 2 and a GC content between 35 and 65% (light green area within square). GOPHER starts at the outermost end of targeted digests, moves towards the center and selects the first *b*_*min*_ usable probes (dark green). Regions for which no usable probes can be selected are depicted in red. (**c**) If *b*_*min*_ usable probes can be placed within each margin of a given digest, the digest is here referred to as balanced. Otherwise, if 2·*b*_*min*_ probes can be placed in both margins but with unequal numbers in the two margins, the digest is referred to as unbalanced. By default, GOPHER selects balanced digests only, and unbalanced digests can be manually selected after viewpoint creation, but if desired users can allow GOPHER to select unbalanced digests if no balanced digest can be found

Capture Hi-C probes must meet certain requirements that are substantially different from the those for standard use cases such as exome sequencing. Note that in this article, we refer to the DNA sequences produced by the sonication step of next-generation sequencing as fragments, and we refer to the DNA sequences produced by restriction digestion as digests. Within Hi-C libraries, interacting sequences are represented by hybrid molecules consisting of two pieces of digests from different genomic locations (Fig. 2a). The sonication step decreases the length of hybrid molecules, typically to around 300–500 bp. Therefore, valid interaction read pairs [26] map largely to the margins of digests adjacent to restriction enzyme cutting sites (Additional file 1: Figure S1). GOPHER takes this into account and places probes only within the margins of digests with a default size of 250 bp.

GOPHER considers alignability as well as GC content of probes (Fig. 2b). The mean k-mer alignability (“Methods” sections) of a probe reflects the average number of sequences in the target genome that are identical with k-subsequences of the probe. It is assumed that a higher k-mer alignability may increase the probability of unspecific cross hybridization of the probe to repetitive genomic sequences and thereby reduce the capture efficiency of the probe. By default, GOPHER discards probes with mean k-mer alignabilities greater than 2; there is a tradeoff between the mean alignability threshold and the number of viewpoints for which probes can be designed, and the threshold can be adjusted by the user (Additional file 1: Figure S2). GOPHER restricts the GC content of selected probes between a lower threshold of 35% and an upper threshold of 65%, but these default thresholds can be adjusted by the user. For each margin of a given targeted digest, GOPHER starts at the outermost ends, moves towards the center and selects the first *b*_*min*_ usable probes. There is no restriction on the overlap between probes, because we reasoned that the sequences directly next to the cutting sites occur most likely within hybrid fragments (Additional file 1: Figure S1). Furthermore, complete tiling of the margins is not an appropriate objective in this case. Therefore, if a margin contains more than one probe, it is often the case that the probes are only shifted by only 1 bp. The parameter *b*_*min*_ denotes the minimum number of probes (baits) necessary to select a digest for enrichment. By default, GOPHER demands that each of the two margins of a digest contain *b*_*min*_ probes; if this is the case, the digest is referred to as balanced. If the user allows unbalanced margins in the Setup tab of GOPHER, then any digest with at least 2·*b*_*min*_ valid probes will be selected. If the two margins do not have equal numbers of probes, then the digest is referred to as unbalanced (Fig. 2c). GOPHER prefers balanced digests because they may be associated with a more even enrichment. However, if it is preferable for the experimental goals to have unbalanced digests rather than no digests at all for difficult sequences, then the user can select unbalanced margins or manually select individual digests after creation of viewpoints.

### Viewpoint creation

**Fig. 3:**
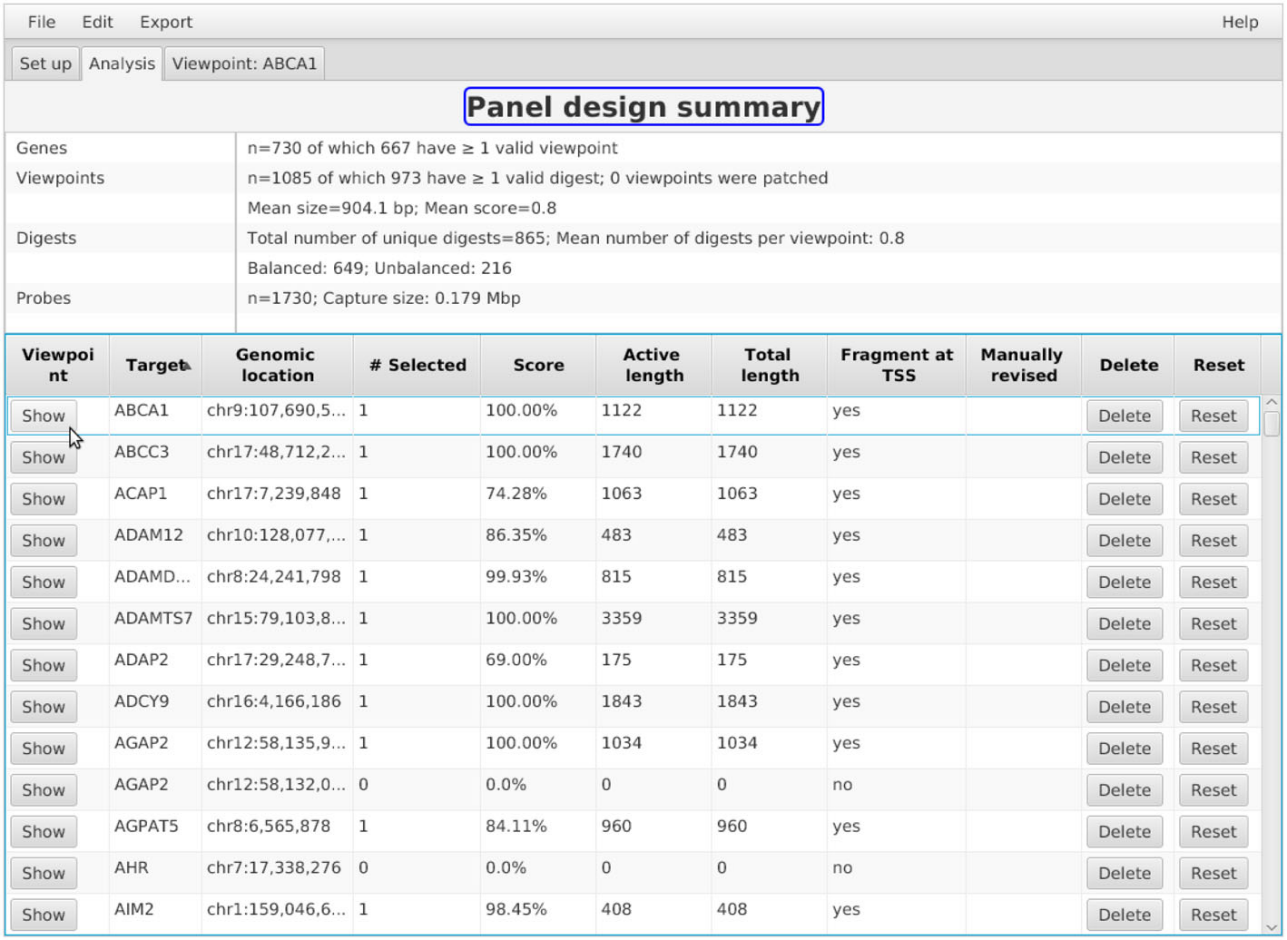
Simple viewpoint creation. Simple viewpoints can be created by clicking on Create viewpoints! after setting of appropriate parameters (Fig. 1c). Upon completion, the Analysis tab will be opened. At the top, summary statistics regarding the design are listed. In this case, GOPHER attempted to create simple viewpoints for 730 genes. GOPHER created at least one valid viewpoint (at least one selected digest) for 667 genes. Note that there are usually more viewpoints than genes, because one viewpoint for each TSS is created. For instance, two viewpoints were created for the gene AGAP2. If the the simple approach is performed without patching, the mean size of viewpoints corresponds to the mean size of digests at TSS. Depending on the selected restriction enzyme, this size may be different from the mean size derived from all digests due to the different base composition in promoter regions. Overlapping viewpoints arising from multiple TSS on given digests lead to redundant digests and associated probes. GOPHER reports only the number of unique digests and does not export redundant probes. The unique digests are further classified as balanced and unbalanced. The number of probes and the capture size, i.e. the total region that is covered by probes, can be used for cost estimation. The table below the summary statistics contains information about individual viewpoints. Each viewpoint can be opened for visual inspection and editing. Manually adjusted adjusted viewpoints will be flagged and can be reset to their original state

Following data preparation and the choice of parameters, the user can click the Create Viewpoints button to cause GOPHER to read the genome sequence and alignability map in order to prepare an *in silico* digest and to evaluate each digest and candidate probe sequence with respect to *k*-mean alignability and GC content. A progress monitor tracks the creation of the viewpoints. Following this, the Analysis tab will be initialized to show a summary of the results and one row for each created viewpoint (Fig. 3). Users can click on individual viewpoints to show Viewpoint editor tabs that will be discussed below.

### Creation of simple viewpoints

**Fig. 4:**
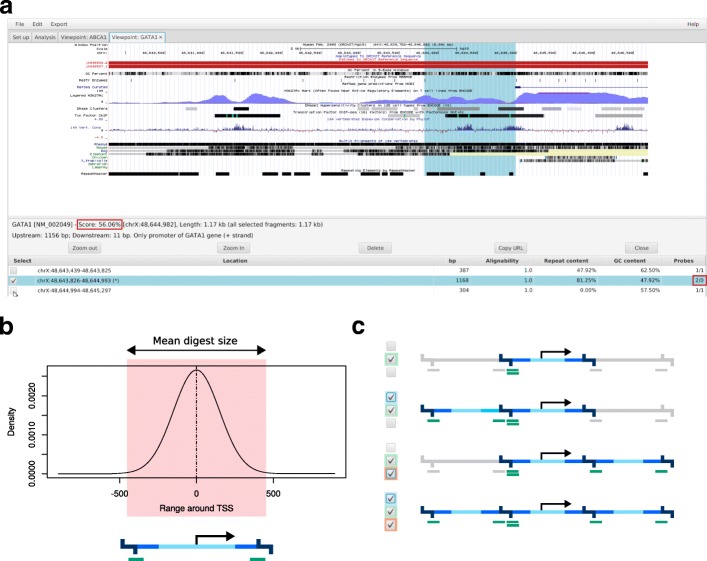
Simple viewpoint creation. (**a**) From the Analysis tab (Fig. 3) each individual viewpoint can be opened in a separate tab for visual inspection. The upper part displays tracks from UCSC’s genome browser and can be used for evaluation and orientation during editing of viewpoints. In this case, the selected digest is not well centered at the TSS. Detailed information about the digest that contains the TSS (marked with an asterisk) and the two adjacent digests are shown below. The indicated information about alignability, GC and repeat content refers to selected probes. Note that in this case the digests containing the TSS is unbalanced due to high GC content at the downstream margin. (**b**) The score for simple viewpoints is close to 1 for digests that are not too short and well centered at the TSS, whereas it is close to 0.5 if the TSS occurs at the outermost ends of digests. Such viewpoints can be easily identified by sorting the viewpoint table in the Analysis tab by score. (**c**) The user can select and deselect each individual digest. For the GATA1 viewpoint shown above, the adjacent downstream digest should be selected in order to center the viewpoint at the TSS

GOPHER’s simple approach is intended for designs with a large number of target regions. In such cases the number of available probes may become a limiting factor. For instance, to capture the human promoters of protein-coding, noncoding, antisense, snRNA, miRNA and snoRNA transcripts about 22,000 *Hind*III restriction fragments (digests) were targeted with two probes each [7, 10]. Only one digest is targeted for each viewpoint; the digest that overlaps the transcription start site (TSS) is chosen if possible (Fig. 4). In many studies, the 6-cutter *Hind*III (∼ 3700 bp) is employed for promoterome-wide investigations, but GOPHER allows a range of 6-cutters and 4-cutters such as *Dpn*II (∼ 430 bp) for different experimental goals. Depending on the cutting motif, some restriction enzymes may display a different distribution of digest sizes near to the transcription start sites. For instance, for *Dpn*II the digests at TSS are on average 900 bp instead of 430 bp. Especially if 4-cutters are used (which tend to generate smaller digests than 6-cutters), we have observed that in some viewpoints, the digest only barely overlaps the actual TSS, with a substantial amount of potentially important regulatory sequence (as judged by the presence of an H3K27Ac peak) being left out (Fig. 4a). GOPHER calculates a score for simple viewpoints that reflects how well the region around given TSS is covered by the associated digest (Fig. 4b). Viewpoints with poor coverage tend to have scores close of 0.5 or less and can be identified via sorting the table in the Analysis tab (Fig. 3). The Viewpoint editor tab allows the user to add additional adjacent digests by selecting the corresponding checkbox (Fig. 4c). With the simple approach, a total of three digests are shown, with the selected digest being in the middle. In some cases, the surrounding digests cannot be chosen because they are too short or no baits can be found which satisfy the chosen GC or alignability constraints. In this case, GOPHER shows “n/a” in red.

### Simple patched viewpoints

**Fig. 5:**
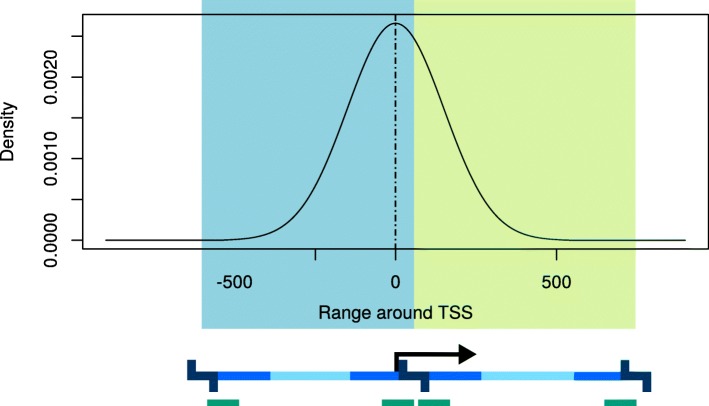
Simple patched viewpoints. In the first run of simple viewpoint creation, only digests that contain the TSS are selected. This may result in viewpoints that are not well centered at the TSS. Such viewpoints can be manually revised by selecting adjacent digests in upstream or downstream direction (Fig. 4c). GOPHER provides an option referred to as “patching“ that automates this process. If the score of a simple viewpoint is below 0.6, GOPHER will add the appropriate adjacent digest if possible

The creation procedure of simple viewpoints may result in viewpoints that are not well centered at the TSS and thus might miss relevant regulatory elements. In such cases adjacent digests can be additionally selected manually, which is time-consuming for larger numbers of viewpoints. Therefore, GOPHER provides the simple patched approach that automates the process of selecting the best digest (Fig. 5). First, simple viewpoints are generated as described above. For viewpoints whose score is less than 0.6, GOPHER tries to add one of the two adjacent digests. GOPHER selects the digest that is closer to the TSS if it satisfies length, alignability, and GC content criteria. After patching, the simple viewpoint score is recalculated, and poor-quality viewpoints can be identified by sorting as for the simple approach.

### Extended viewpoints

Some published CHC studies target all promoters of the genome by placing single probes at the the outermost ends of TSS-containing *Hind*III restriction fragments [7, 8, 10, 27]. The tools CapSequm [6, 28] and HiCapTools [25] can be used generate probes for this class of experiment, and GOPHER’s simple and simple-patched approaches are mainly intended for this setting. On the other hand, some CHC studies targeted only a few hundred promoters for enrichment using the 4-cutter *Dpn*II [6, 29]. These studies provide higher resolution and deeper coverage at the viewpoints.

**Fig. 6:**
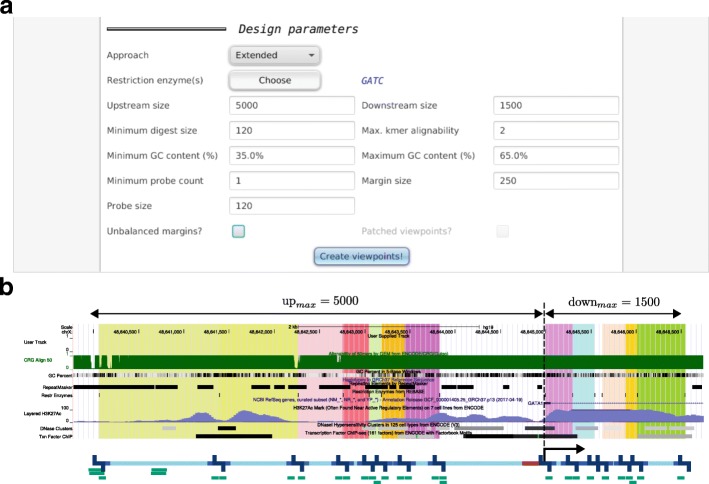
Extended viewpoints. The extended approach is intended for in-depth analysis of a few hundred predefined genes of interest. (**a**) Instead of restricting the view only to digests containing the TSS, minimal distances to the TSS in up and downstream directions can be specified by the user. All digests that overlap the this range and satisfy the design parameters are selected. (**b**) Extended viewpoint for *GATA1*. The Copy URL button of the Viewpoint editor tab can be used to visualize viewpoints in UCSC’s genome browser. Just as for the embedded view within GOPHER, selected digests are indicated by colored boxes in the background. This representation has the advantage that additional custom tracks can be loaded in order to evaluate viewpoints. In this case the track for alignability is displayed (green). For this track, the alignability is standardized to values greater or equal 0 and lower or equal 1. For example, a value of 1/2 means that the corresponding k-mer occurs 2 times in the genome. The unbalanced digest at the TSS is not selected but can selected manually or directly during creation of viewpoints, if *Unbalanced margins* is set

We developed the extended approach to enable probe design for the latter class of experiment. GOPHER will select all digests that are located within or overlap with the window specified by upstream size and downstream size and that display a valid size, mean k-mer-alignability, and GC content. GOPHER calculates an empirical viewpoint score for extended viewpoints that can be used in the same way as the score for the simple and simple-patched approaches to rank the viewpoints and if desired prioritize viewpoints with low scores for manual inspection. Similar to the score for simple viewpoints, viewpoints that are well covered receive a good score, and selected digests near to the TSS contribute more to the score than others (“Methods” section; Fig. 6).

### Non-TSS applications

GOPHER can be used to target genomic regions in which GWAS hits are embedded. Users should generate a BED6 file [30] with the genomic coordinates of the GWAS hits and adjust the parameters such as restriction enzyme and probe criteria according to experimental goals. GOPHER can also be used to generate probes to “tile” larger genomic regions by choosing the extended approach with a BED file that contains a position at the center of each desired genomic region. The size of the genomic region is controlled by adjusting the Upstream and Downstream size in GOPHER’s Setup tab.

### GOPHER features

GOPHER shares a record of the project file in the user’s home directory, so that users can return to a probe design project at multiple occasions after saving the current results via the File menu. If desired, the project file can also be exported and shared with other GOPHER users. GOPHER outputs a number of files, including a documentation of the current project and results, a BED file with the chromosomal location of the selected digests and a probe file that can be used for ordering probes from commercial suppliers. We note that if two viewpoints share the same selected digest, the digest margins and probes that are output as input for probe design are made unique. The same is true for exported probes. GOPHER also can export a digest file with data on each of the in silico digests including information on GC content, alignability, length, and whether a probe was chosen or not. This file can be used to provide this data to downstream analysis programs for normalization or interaction calling. Table 2 provides an overview of GOPHER’s output files.

**Table 2:** GOPHER output files: Seven files can be exported from GOPHER via the Export menu. These files can be used for downstream analyses, documentation, visualization, and ordering of probes. The file prefix <PREFIX> corresponds to the name of the project. The prefix of the digest file additionally contains the tag for the genome build

Filename	Description
<PREFIX>_DigestedGenome.txt	*Content of lines:* Coordinates, length, GC and repeat content, indication of selection, number of probes for each digest.
	*Purpose:* Can be used for downstream analyses.
<PREFIX>_viewPoints.tsv	*Content of lines:* Gene symbol, coordinates, UCSC custom URL, number of selected digests, score, length, indication whether digest with TSS is selected.
	*Purpose:* Documentation and sharing of results.
<PREFIX>_uniqueTargetDigests.bed	*Content of lines:* Coordinates and names of targeted digests. Duplicates arising from overlapping viewpoints are removed.
	*Purpose:* Probe design with external tools.
<PREFIX>_uniqueTargetDigestMargins.txt	*Content of lines:* Coordinates and names of margins of targeted digests.
	*Purpose:* Probe design with external tools.
<PREFIX>_allTracks.bed	*Content of lines:* Contains multiple sections for viewpoints, digests, margins, and probes.
	*Purpose:* Can be uploaded as multiple custom tracks to UCSC.
<PREFIX>_ProbeFile.bed	*Content of lines:* Coordinates and names of probes.
	*Purpose:* Ordering of probes.
<PREFIX>_agilentProbeFile.txt.zip	*Content of lines:* Coordinates, names, and sequences of probes.
	*Purpose:* Ordering of probes.

The allTracks.bed output file can be loaded as a custom track into UCSC’s genome browser [30]. This file contains five tracks: Genomic positions (e.g., the TSS), Viewpoints, Restriction fragments, Target regions and Probes (Additional file 1: Figure S3). The viewpoints are displayed in different grayscales that reflect the scores of viewpoints (black for viewpoints with scores of 100%). Restriction fragments and target regions (margins of restriction fragments) are depicted in blue. Probes are displayed in different grayscales that reflect the mean k-mer-alignabilities (MKA) of probes (black for a MKA of 1 and gray for higher values). Currently, alignability maps for mm9 and hg19 are available within UCSC but not for mm10 and hg38.

## Discussion

In CHC projects published to date, probes have been designed by the online tool CapSequm, by various (undocumented) scripts, and some have been manually designed or revised [6, 10–19, 23, 29]. In our experience, CHC probe design is time consuming and hard to document, and different studies have used different viewpoint and probe definitions without the motivation behind the differences being explicitly stated. For example, for the promoter of *Prrx1* two different viewpoints (Additional file 1: Figure S4) were used in two different studies [6, 29].

The current work presents a formalization of the criteria used to evaluate CHC probes, and provides an easy to use Java desktop application that implements three strategies for CHC probe design. GOPHER’s simple approach is similar to the probe design strategy taken in previous publications that employ CHC to investigate all promoters, with one probe being placed at each margin of single digests that overlap TSS. This approach is suitable for large numbers of target genes, e.g., the promoterome, and is often used with a six-cutter to obtain relatively large enriched digests. If four-cutters are used, in general many target genes will be discarded because digests are too short or not well centered at the TSS. GOPHER’s simple-patched approach is an extension of the simple approach that intends to improve coverage of viewpoints, which is accomplished by adding adjacent digests in upstream or downstream direction. GOPHER’s extended approach is intended mainly for focused investigations of smaller gene sets; here, we recommend the use of a four-cutter to obtain higher resolution, which together with GOPHER’s default viewpoint size (5000 bp upstream and 1500 bp downstream) will tend to generate viewpoints with 5–15 enriched digests.

GOPHER provides a series of features that allow users to visualize and edit viewpoints, and also outputs a series of files that are useful for a number purposes. For instance, an appropriately formatted probe file can readily be used for ordering, or a file containing the size, GC-content, alignability, and number of probes for individual digests can be used for normalization of interaction counts [31, 32]. Finally, it is also possible to export entire projects to files that can be shared between GOPHER users.

## Conclusion

GOPHER allows probes for capture Hi-C viewpoints to be created within a few hours according to one of three different design approaches using clear and consistent rules. The graphical user interface allows post hoc inspection and editing of individual viewpoints. GOPHER will enable a wider range of researchers to employ CHC by providing an easy-to-use Java desktop application for CHC probe design. Source code, precompiled executables and detailed documentation are available on the GOPHER GitHub page at https://github.com/TheJacksonLaboratory/Gopher.

## Methods

### Implementation

GOPHER is a desktop Java application written using the JavaFX library for designing graphical user interfaces. GOPHER requires Java 1.8. The source code of GOPHER can be downloaded from the GOPHER GitHub page:

https://github.com/TheJacksonLaboratory/Gopher

From here, users can also download a precompiled application. Detailed documentation is available on a readthedocs site. GOPHER is freely available for academic use.

### Data and data preparation

GOPHER allows the user to download all necessary data directly from the GOPHER application prior to generating probes. GOPHER downloads the genome sequence, transcript annotations, and alignability map for the human (hg19 or hg38) and mouse (mm9 or mm10) genomes. Default values are provided for all relevant parameters, and the user can change parameters directly in the application window (Table 1).

### In silico digestion of the target genome

GOPHER downloads genome files from the UCSC Genome Browser Database [30]. It extracts this file (which is downloaded as a g’zipped file) and combines the individual chromosome files into a single file, which it writes to disk (for instance, the file for the human hg38 genome would be named hg38.fa). It then uses the HTSJDK library, a Java API for high-throughput sequencing data (HTS) formats that is part of the SAMtools suite [33], to create a FASTA index file. During the creation of viewpoints, GOPHER utilizes functions of HTSJDK together with these files to identify all digests on the basis of the enzyme or enzymes chosen by the user.

### Mean k-mer alignability of probes

For mouse mm9 and human hg19 genome builds, we used the CRG Alignability tracks for a k-mer size of 50 that are available as default tracks in UCSC’s genome browser (wgEncodeEM002940 and wgEncodeEH000320). The alignability is standardized to values in the range [0,1]. For instance, 0.25 means that the corresponding k-mer occurs 4 times within the entire genome. These tracks were created in the course of the ENCODE project [34] using the mappability program of the GEM suite (GEnome Multitool) [35]. For the mm10 and hg38 genome builds, no alignability tracks were available. Therefore, we generated new tracks using GEM version 1.7.1. In accordance with the existing maps, we allowed at most 2 mismatches (-m 2). The index was built on all chromosomes including non-canonical chromosomes. The output of GEM was converted to bedGraph format using utility programs of the kentUtils tool suite [36]. Lines for non-canonical chromosomes were removed and compressed files were uploaded to ftp://ftp.jax.org/robinp/GOPHER/alignability_maps. GOPHER automatically downloads the file as required for the genome build being analyzed.

Within GOPHER, the alignability map *p*↦*a*_*k*_(*p*) indicates how often the k-mer starting at position *p* occurs in the entire genome. For instance, *a*_50_(42)=2 means that the 50mer starting at position 42 occurs two times in total. GOPHER uses binary search for efficient retrieval of all k-mer alignabilities within a given target region which can be used for fast calculation of mean k-mer alignabilities of digests. For a probe of length *l* starting at position *p*, the mean k-mer alignability is calculated as follows:

1 $$ a_{k}(p)=\frac{1}{l-k+1} \cdot \sum\limits_{i=0}^{l-k}{a_{k}(p+i)}.  $$

By default, probes with a mean k-mer alignability above 2 are rejected, but users can adjust this threshold value.

### Mean G/C content of probes

GOPHER uses the HTSJDK library to retrieve the sequences of candidate digests and probes and counts the number of G, C, A, and T bases in order to calculate the GC content. By default, probes with a GC content of between 35 and 65% are accepted, but users can adjust these threshold values.

### Score for simple viewpoints

Ideally, the selected digest of a simple viewpoint is centered at the TSS and not too short. In order to capture this intuition, we estimate the average size $\bar {\mu _{D}}$ from all digests in the genome and use a normal distribution with a mean *μ*=0 that corresponds to the TSS and a standard deviation $\sigma =1/6 \cdot \bar {\mu _{D}}$. The score is then calculated as the area under the curve of the probability density function within the range that is covered by the digest containing the TSS. The score can take on values between 0 and 1, and digests that are not well centered at the TSS will be assigned a score close to 0.5.

### Score for extended viewpoints

The score for extended viewpoints is very similar to the score for simple viewpoints but it allows asymmetric length ratios for the targeted regions upstream and downstream of the TSS. Given the user-specified maximum allowed upstream and downstream distances from the TSS (Table 1), an extended viewpoint can be seen as a set of selected digests that overlap the specified range [TSS−up_*max*_,TSS+down_*max*_]. For a good viewpoint, the selected digests should cover as many positions as possible within the specified range, especially near the TSS.

To calculate the empirical score, we model the coverage of a viewpoint and its associated upstream and downstream sizes (5000 bp and 1500 bp by default) as based on normal distributions with mean at the transcription start site (or other central genomic position) and standard deviations calculated as 1/6 times the upstream or downstream size. Since 6 standard deviations cover ∼ 99.999% of the total probability, if chosen digests cover all of the upstream space they will add 0.5 to the total probability and similarly with the downstream digests. In general, a digest will contribute more to the total probability the closer it is to the TSS. The probability is multiplied by 100 and the score is reported as a percentage.

## Availability and requirements

- **Project name:** GOPHER: Generator Of Probes for capture Hi-C Experiments at high Resolution
- **Project home page:** https://github.com/TheJacksonLaboratory/Gopher
- **Operating system:** Platform independent
- **Programming language:** Java
- **Other requirements:** Java 1.8
- **License:** JAX
- **Any restrictions to use by non-academics:** License needed

## Additional file

Additional file 1 Supplementary figures for the distribution of mapped Hi-C interaction read pairs within digests, an exploration of alignability thresholds, heterogeneous definitions of viewpoints and explanation of GOPHER’s allTracks.bed file. (PDF 1430 kb)

## Abbreviations

3C: Chromosome conformation capture
CHC: Capture Hi-C
HTS: High-throughput sequencing
GEM: Genome multitool
GOPHER: Generator of probes for capture hi-c experiments at high resolution
GUI: Graphical user interface
GWAS: Genome-wide association study
MKA: Mean k-mer-alignability
SNP: Single nucleotide polymorphism
TAD: Topologically associating domain
TSS: Transcription start site
