# Supplementary material for: GOPHER: Generator Of Probes for capture Hi-C Experiments at high Resolution
Source: BMC Genomics. 2019 Jan 14;20:40. doi: 10.1186/s12864-018-5376-4 (PMC6332836; doi:10.1186/s12864-018-5376-4)
Supplement: Supplementary file 1 — Supplementary figures for the distribution of mapped Hi-C interaction read pairs within digests, an exploration of alignability thresholds, heterogeneous definitions of viewpoints and explanation of GOPHER’s allTracks.bed file. (PDF 1430 kb) [file 12864_2018_5376_MOESM1_ESM.pdf]

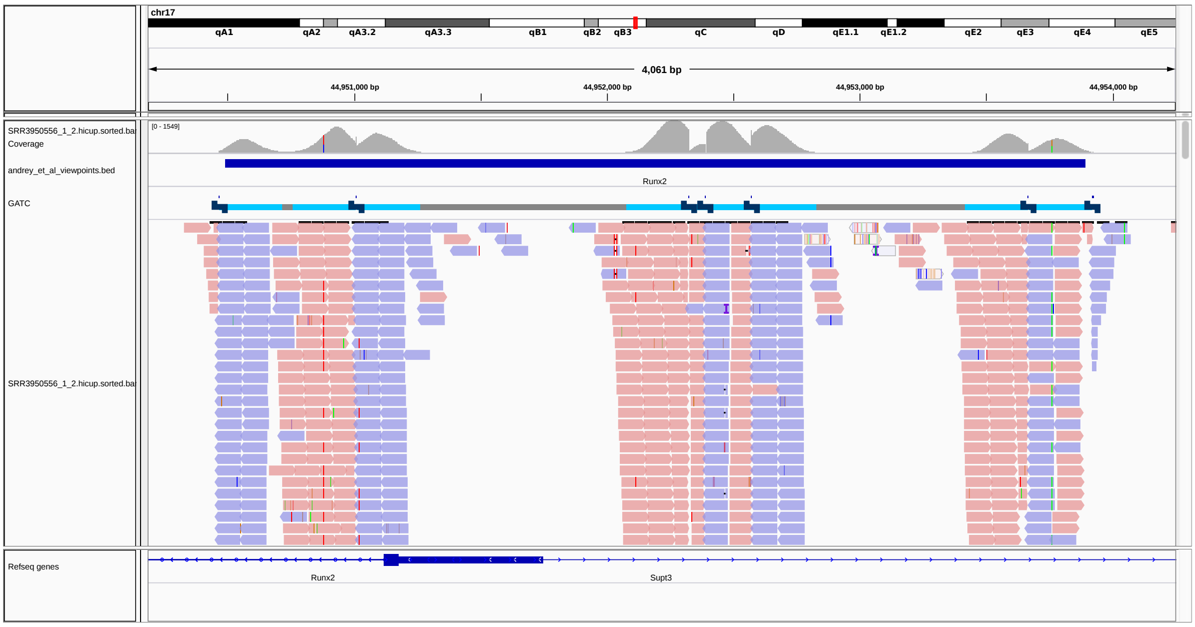

Figure S1. Distribution of valid interaction read pairs within digests. This screenshot was taken from the Integrative Genomic Viewer (IGV) [1]. It shows Capture-C reads (`SRR3950556`) mapped to a viewpoint at the promoter of the `Runx2` gene in the mouse mm9 genome [2]. The reads were mapped and filtered for artifacts using the HiCUP pipeline [3]. HiCUP's unique valid interaction read pairs map only to the margins of digests (light blue bars, 250 bp), even though for this experiment the entire viewpoint was tiled with probes without taking into account cutting sites of the restriction enzyme DpnII.

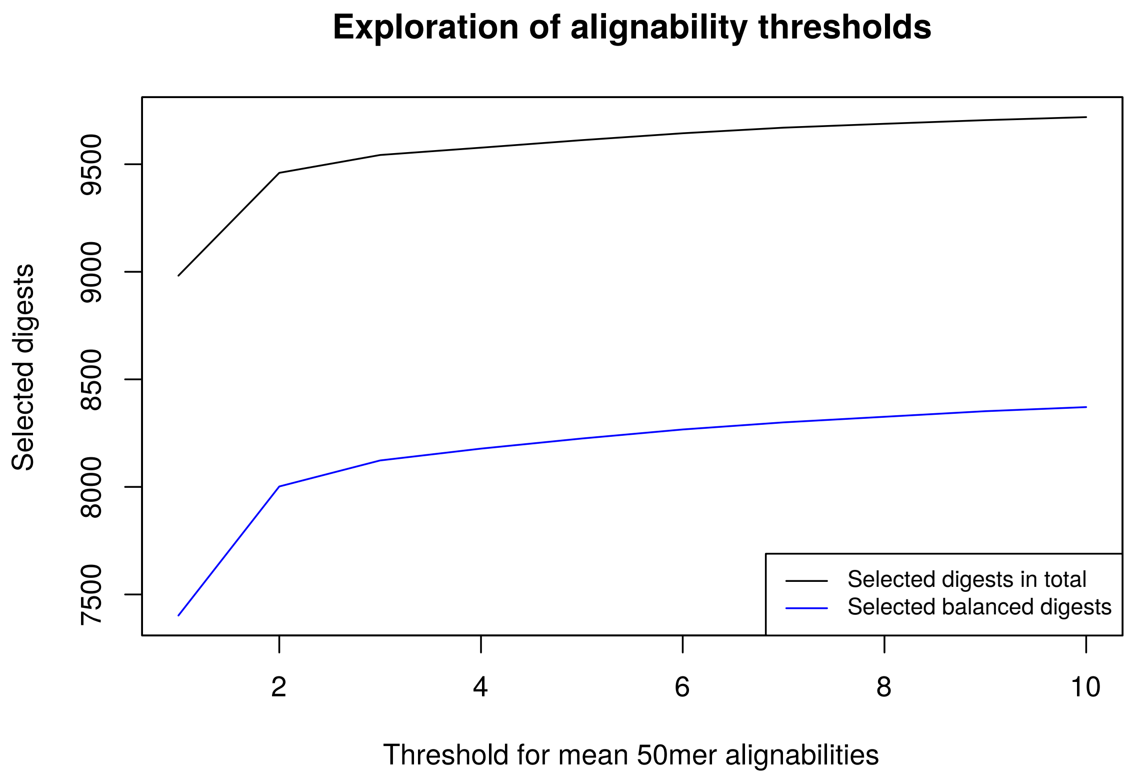

Figure S2. Exploration of different alignability thresholds. The GOPHER software was used to create extended viewpoints for 731 genes using $b_{min} = 3$ and `Unbalanced margins?` set to `True`. The creation of viewpoints was repeated using different settings of `Max kmer alignability` ranging from 1 to 10. The plot shows the number of selected digests for different alignability thresholds. The total number of selected digests (black) is the number of balanced (blue) plus the number of unbalanced selected digests.

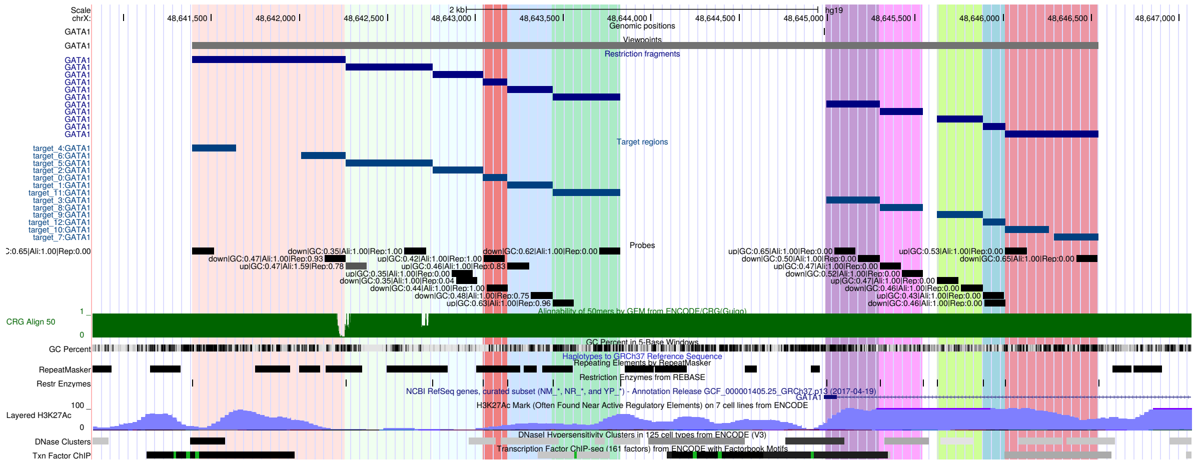

Figure S3. GOPHER's AllTracks BED file loaded as custom track in UCSC's genome browser. The BED file <PREFIX>_allTracks.bed can be exported via the export menu and loaded as a custom track into UCSC's genome browser [4]. This file contains five tracks: Genomic positions (in this case the TSS), Viewpoints, Restriction fragments, Target regions and Probes. The viewpoints are displayed in different grayscales that reflect the scores of viewpoints (black for viewpoints with scores of 100%). Restriction fragments and target regions (margins of restriction fragments) are depicted in blue. Probes are displayed in different grayscales that reflect the mean kmer-alignabilities (MKA) of probes (black for a MKA of 1 and gray for higher values). Next to each probe the corresponding GC content, MKA and repeat content are shown. In this case there is only probe has a MKA greater than 1 (upstream margin of the second digest). This can be retraced using the alignability map shown below (green). Note that for 50mers and probes with 120 bp only the alignability scores of the first 70 bp have to be taken into account for calculation of the MKA. Therefore, the last 50 bp of probes with MKA= 1 may overlap regions with regions that have scores less than 1. Alignability maps for mm9 and hg19 are available within UCSC but not for mm10 and hg38.

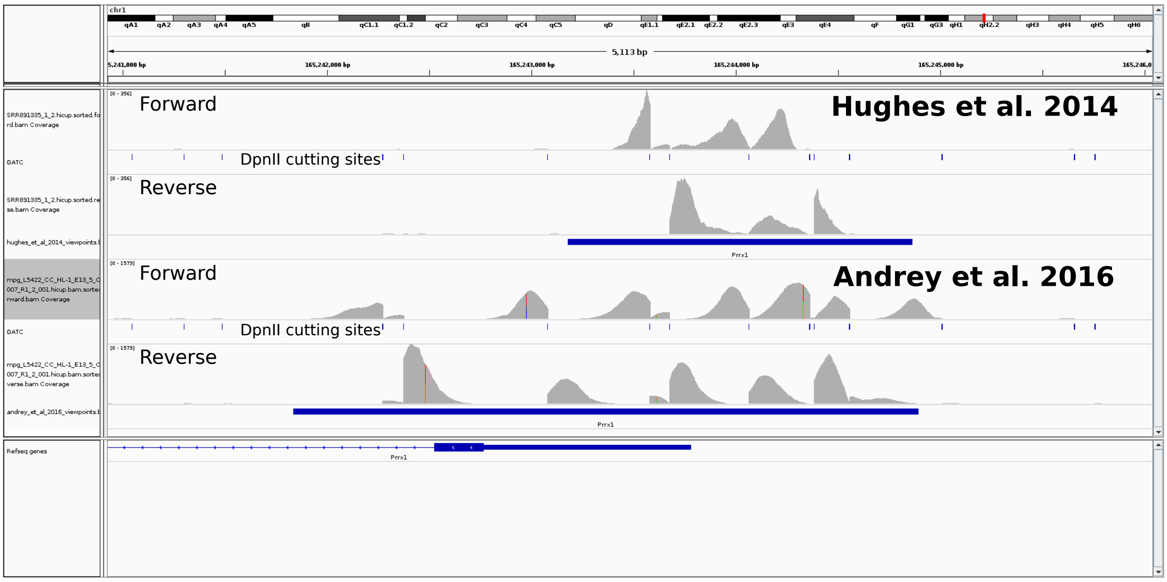

Figure S4. Heterogeneous definitions of viewpoints. In two studies [2, 5] different viewpoints were defined for the same promoter region of the *Prrx1* gene.

References (Online Supplementary Material)

[1] Robinson, J.T., Thorvaldsdóttir, H., Winckler, W., Guttman, M., Lander, E.S., Getz, G., Mesirov, J.P.: Integrative genomics viewer. Nature biotechnology **29**(1), 24–26 (2011). doi:10.1038/nbt.1754

[2] Andrey, G., Schöpflin, R., Jerković, I., Heinrich, V., Ibrahim, D.M., Paliou, C., Hochradel, M., Timmermann, B., Haas, S., Vingron, M., Mundlos, S.: Characterization of hundreds of regulatory landscapes in developing limbs reveals two regimes of chromatin folding. Genome research, 213066–116 (2016). doi:10.1101/gr.213066.116

[3] Wingett, S., Ewels, P., Furlan-Magaril, M., Nagano, T., Schoenfelder, S., Fraser, P., Andrews, S.: HiCUP: pipeline for mapping and processing Hi-C data. F1000Research **4**, 1310 (2015). doi:10.12688/f1000research.7334.1

[4] Casper, J., Zweig, A.S., Villarreal, C., Tyner, C., Speir, M.L., Rosenbloom, K.R., Raney, B.J., Lee, C.M., Lee, B.T., Karolchik, D., Hinrichs, A.S., Haeussler, M., Guruvadoo, L., Navarro Gonzalez, J., Gibson, D., Fiddes, I.T., Eisenhart, C., Diekhans, M., Clawson, H., Barber, G.P., Armstrong, J., Haussler, D., Kuhn, R.M., Kent, W.J.: The ucsc genome browser database: 2018 update. Nucleic Acids Research **46**, 762–769 (2018). doi:10.1093/nar/gkx1020

[5] Hughes, J.R., Roberts, N., McGowan, S., Hay, D., Giannoulatou, E., Lynch, M., De Gobbi, M., Taylor, S., Gibbons, R., Higgs, D.R.: Analysis of hundreds of cis-regulatory landscapes at high resolution in a single, high-throughput experiment. Nature Genetics **46**(2), 205–212 (2014). doi:10.1038/ng.2871
